# Prolonged SARS-CoV-2-RNA Detection from Nasopharyngeal Swabs in an Oncologic Patient: What Impact on Cancer Treatment?

**DOI:** 10.3390/curroncol28010083

**Published:** 2021-02-08

**Authors:** Anna Ferrari, Marco Trevenzoli, Lolita Sasset, Elisabetta Di Liso, Toni Tavian, Lucia Rossi, Eugenia Di Meco, Anna Maria Cattelan

**Affiliations:** 1 Infectious Diseases Unit, Azienda Ospedale Università di Padova, 35128 Padova, Italy; marco.trevenzoli@aopd.veneto.it (M.T.); lolita.sasset@aopd.veneto.it (L.S.); edm1984@gmail.com (E.D.M.); annamaria.cattelan@aopd.veneto.it (A.M.C.); 2 Medical Oncology 2, Istituto Oncologico Veneto IRCCS, 35128 Padova, Italy; elisabetta.diliso@ioveneto.it; 3 Department of Medicine, Geriatric Clinic, Università di Padova, 35128 Padova, Italy; toni.tavian@gmail.com; 4 Microbiology Department, Azienda Ospedale Università di Padova, 35128 Padova, Italy; lucia.rossi@aopd.veneto.it

**Keywords:** non-small cell lung cancer, SARS-CoV-2 RNA, COVID-19, viral culture, cancer treatment

## Abstract

The pandemic of SARS-CoV-2 is a serious global challenge affecting millions of people worldwide. Cancer patients are at risk for infection exposure and serious complications. A prompt diagnosis of SARS-CoV-2 infection is crucial for the timely adoption of isolation measures and the appropriate management of cancer treatments. In lung cancer patients the symptoms of infection 19 may resemble those exhibited by the underlying oncologic condition, possibly leading to diagnostic overlap and delays. Moreover, cancer patients might display a prolonged positivity of nasopharyngeal RT-PCR assays for SARS-CoV-2, causing long interruptions or delay of cancer treatments. However, the association between the positivity of RT-PCR assays and the patient’s infectivity remains uncertain. We describe the case of a patient with non-small cell lung cancer, and a severe ab extrinseco compression of the trachea, whose palliative radiotherapy was delayed because of the prolonged positivity of nasopharyngeal swabs for SARS-CoV-2. The patient did not show clinical symptoms suggestive of active infection, but the persistent positivity of RT-PCR assays imposed the continuation of isolation measures and the delay of radiotherapy for over two months. Finally, the negative result of SARS-CoV-2 viral culture allowed us to verify the absence of viral activity and to rule out the infectivity of the patient, who could finally continue her cancer treatment.

## 1. Introduction

Severe acute respiratory syndrome coronavirus 2 (SARS-CoV-2) pandemic is a complex global challenge affecting millions of people worldwide [1]. Cancer patients are at high risk for serious complications and the infection itself may compromise the continuation of cancer therapies and supportive care [2,3,4]. Moreover, the frequent visits to the hospital for treatment and monitoring represent for cancer patients a major risk factor for exposure to SARS-CoV-2 [5]. In order to optimize the cancer care pathway and improve the clinical outcomes during the pandemic, a large, international and multidisciplinary consortium reviewed and discussed the clinical evidence in cancer management. In this current situation, the risk of infection must be individualized taking into consideration the primary tumor type, stage, age, and sex. In addition, cancer treatment must be patient tailored [6]. Thus far, the gold standard test for the diagnosis of COVID-19 has been the RT-PCR test performed on nasopharyngeal swabs or other upper respiratory tract specimens. However, a “positive” PCR result reflects the detection of viral RNA and does not necessarily prove the presence of active viral replication. To ascertain the infectivity of RT-PCR positive patients, several diagnostic methods such as the viral culture, the viral RNA measured by the cycle threshold (Ct), the antigen rapid test, and the detection of sub-genomic RNAs (sg-RNAs) have been recently introduced in clinical care. All these tests, when correlated with the time course of disease, may help define the contagiousness of patients diagnosed with SARS-CoV-2 infection [7,8,9,10]. We describe the case of a cancer patient whose palliative radiotherapy treatment was delayed because of the prolonged positivity of RT-PCR assays for SARS-CoV-2 on nasopharyngeal swabs, despite the absence of acute signs of infection. Eventually, the negative viral culture result allowed the physicians to continue the treatment.

## 2. Case Report

On 1 April 2020, a 72-year-old Caucasian woman affected by non-small cell lung cancer of the upper right lobe, was referred to the Istituto Oncologico Veneto (IOV) of Padua, Italy, for the acute onset of progressive shortness of breath and dry cough. Her past medical history was relevant for glaucoma, depression, and Parkinson disease. The patient had undergone a right upper lobe lung resection with ilo-mediastinic lymph node dissection in 2015; the histo-pathological examination revealed an EGFR exon19 deletion mutated adenocarcinoma. After four courses of adjuvant chemotherapy (cisplatin plus gemticitabine), due to disease progression, in 2018 targeted therapy with osimertinib was started with partial response according to RECIST (response evaluation criteria in solid tumors version 1.1).

At hospital admission, the physical examination revealed moderate inspiratory retraction and diffuse inspiratory wheezing; the body temperature was 36.8 °C; heart rate 68 bpm; blood pressure 125/80 mm Hg; respiratory rates 25 per minute; oxygen saturation 98% with 3 L/min oxygen supplement. Her ECOG PS (Eastern Cooperative Oncology Group performance status) was 2.

**Figure 1:**
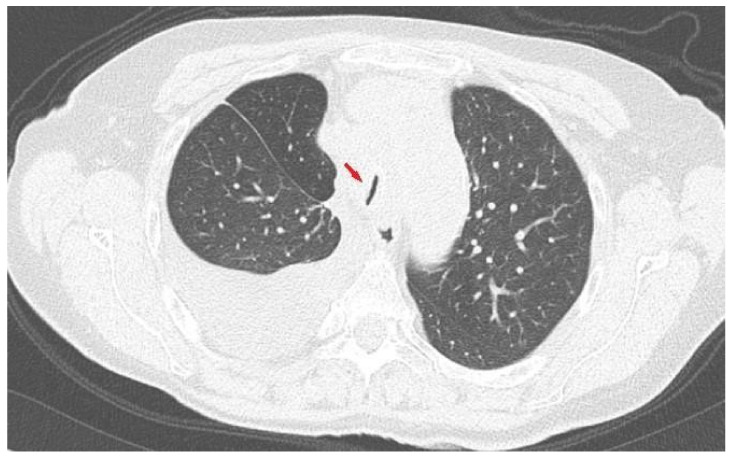
Chest computed tomography (CT) scan: axial lung image showing huge reduction (arrow) of the trachea lumen (2 mm) due to mediastinal pathological tissue.

Laboratory investigations were all within the normal range, including lactate dehydrogenase (LDH), C-reactive protein (CRP), and procalcitonin. The nasopharyngeal swab for the detection of SARS-CoV-2 RNA by RT-PCR [11] was negative. A computed tomography (CT) scan of the thorax showed progressive disease in the superior mediastinum with significant reduction of the tracheal lumen (Figure 1). Palliative mediastinal radiotherapy was planned in order to reduce the tracheal compression, and methylprednisolone 40 mg bid was initiated obtaining a slight reduction of dyspnea.

During the hospital stay, the patient was exposed to an asymptomatic SARS-CoV-2 virus carrier. On 6 April 2020, she underwent screening nasopharyngeal swab for detection of SARS-CoV-2, which was positive. The scheduled radiotherapy was postponed and the patient was transferred to the infectious diseases unit. At admission she presented with mild inspiratory retraction but all the vital signs were normal. Blood examinations showed a moderate elevation of CRP (91 mg/L, normal value 0–6). Because of the high-risk condition, she was treated with the therapy currently recommended: hydroxychloroquine (HCQ) (400 mg bid for 24 h, then 200 mg bid) and azithromycin (AZI) (500 mg qd) [12]. Both drugs were stopped after two days because of QTc prolongation. Tapered steroidal therapy, osimertinib, and subcutaneous enoxaparin were continued.

**Figure 2:**
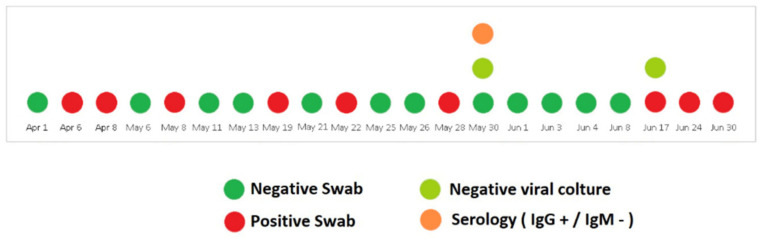
Timeline of SARS-CoV-2 nucleic acid testing results from nasopharyngeal swabs along with the anti-SARS-CoV-2 serology and viral culture testing.

The patient did not develop any coronavirus disease 2019 (COVID-19)-related symptoms, but nasopharyngeal swabs were persistently positive for SARS-CoV-2 for over one month. On 13 May after two consecutive negative swabs, she was transferred to the oncology ward to start radiotherapy treatment. Her ECOG PS was 3. Unfortunately, on May 19, the nasopharyngeal swab for SARS-CoV-2 RNA was positive. Once again the radiotherapy was postponed and the patient was isolated in the infectious diseases ward. The clinical conditions remained stable and the nasopharyngeal swabs were repeated at regular intervals with alternating results (Figure 2). On 30 May, a viral culture with Vero cells [13] was performed and resulted negative. The same result was achieved also 15 days later, despite the occurrence of new positive swabs.

Interestingly, serological tests showed the presence of high levels of anti-SARS-CoV-2 IgG [75,400 kAU/L (Positive: >1100 kAU/L)] and high titers persisted during the whole observation period; IgM title was persistently negative (Figure 2).

On the basis of these results, a single-fraction radiotherapy was performed on 4 June and the patient continued her regular follow-up with no need of oxygen supplementation. The last total body CT scan performed in July 2020 confirmed stable disease.

## 3. Discussion

SARS-CoV-2 pandemic caught clinicians and health authorities unprepared to manage cancer patients. Diagnostic and treatment guidelines were lacking at the beginning of the outbreak. Clinicians were challenged to manage COVID-19 disease with clinical characteristics which might be partly masked or confounded by coexisting cancer conditions. Moreover, the risk of nosocomial transmission of SARS-CoV-2 and the need for isolation contributed to the risk of progression of the malignant disease because of cancer treatments’ delays [14,15,16].

After the detection of SARS-CoV-2 our patient did not show any acute signs of infection and her symptoms could be attributed to her lung cancer. However SARS-CoV-2 swabs remained positive for more than 90 days, showing alternating results: in five occasions they turned out positive after one or two consecutive negative tests. It has been described that, after clinical resolution, some COVID-19 patients may experience a prolonged nucleic acid conversion [17,18]. Xiao and colleagues, from Wuhan, reported 21.4% of patients experiencing a “turn positive” of nucleic acid detection by RT-PCR test after two consecutive negative results [18]. In addition, it is still to be determined whether these patients are able to transmit the infection. However, relevant recent studies have shown that COVID-19 patients with mild-to-moderate illness are highly unlikely to be infectious longer than 10 days after symptom onset [19]. The molecular detection of SARS-CoV-2 by RT-PCR is a rapid and highly sensitive diagnostic method, but its ability to determine the duration of infectivity of patients is quite limited [20]. Viral culture is a more accurate tool to demonstrate the “in vitro” infectivity, giving surrogate information on viral transmission [9]. It is a complex diagnostic tool that needs expertise and biosafety level 3 laboratories, but it could be of crucial importance to define viral transmission and the infectivity of patients in particular conditions, such as the oncological setting [21]. Aside from viral culture, other diagnostic methods such as the threshold cycle (Ct) of RT-PCR and antigen-tests have been reported to be able to determine patients’ contagiousness. It has been demonstrated that infectivity is significantly reduced when Ct values are >24; for every unit increase in Ct, the odds ratio for infectivity decreases by 32%, suggesting that Ct values >24, along with the duration of symptoms >8 days may be used in combination to rule out patients’ contagiousness. Similar results are reported by La Scola et al. who showed a strong correlation between Ct value and sample infectivity in a cell culture model. Based on their data, they inferred that patients with Ct values equal or above 34 do not excrete infectious viral particles [22]. Rapid antigen tests may also have a role in this setting. They have a lower sensitivity compared to the standard RT-PCR test, but may be sensitive enough to detect cases with a high viral load (i.e., low RT-PCR cycle threshold (Ct) value <25), which likely account for a significant proportion of transmissions [8,23]. In addition, as reported in 68 respiratory specimens from 35 COVID patients in Hong Kong, the detection of subgenomic viral RNA (sgRNA) strongly correlated with the presence of active virus replication, with sgRNA being rarely detectable 8 days after the onset of the illness [7]. On the contrary, there is no consensus yet on the significance of the dynamic profile of SARS-CoV-2-specific antibody response. In our case the persistent positivity of RT-PCR assays coexisted with a strong serologic response, and none of the diagnostic procedures could prove or rule out the infectivity of the patient [24]. Unfortunately, in the first half of 2020, when our patient was hospitalized, viral load estimation by threshold cycle of RT-PCR and antigen-based tests were still under investigation, and the detection of sub-genomic RNAs was not available. Furthermore, the World Health Organization guidelines for releasing COVID-19 patients from isolation (i.e., ten days after symptom onset and at least three additional days without symptoms) were not yet amended. Nevertheless these guidelines do not specifically address immunocompromised individuals [25].

An Italian group has recently proposed an algorithm for the management of oncologic patients who need radiation therapy. Their recommendation is to delay or discontinue the radiation treatment in symptomatic and asymptomatic patients diagnosed with COVID-19 and to carefully start the treatment only when the patients are declared no longer exhibiting infection by the infectious diseases specialist [26].

In line with this recommendation we could discontinue hospital isolation owing to the repeatedly negative cell culture results, which allowed us to verify the absence of viral activity despite the persistent positivity of nasopharyngeal swabs. In this way the patient could finally undergo palliative radiotherapy for airway obstruction with a delay of eight weeks from the initial scheduled time. Although it is unlikely that the delayed palliative radiotherapy affected the short-term poor prognosis, it surely prolonged the length of hospitalization and negatively affected the patient’s quality of life.

## 4. Conclusions

This clinical case emphasizes the multifaceted and challenging approach to SARS-CoV-2 infection in the oncological setting. Clinicians must protect the vulnerable cancer population from a potentially severe infection without jeopardizing cancer care and support. The development of diagnostic tests able to determine the activity of the disease and patients’ infectivity may contribute to the fight against SARS-CoV-2, limiting the number of COVID-19-related deaths and the excess indirect mortality and morbidity in patients suffering from cancer.

## References

[1] European Centre for Disease Prevention and Control (2020). COVID-19 Situation Update Worldwide, as of 12 October 2020.

[2] Tian J., Yuan X., Xiao J., Zhong Q., Yang C., Liu B., Cai Y., Lu Z., Wang J., Wang Y. (2020). Clinical characteristics and risk factors associated with COVID-19 disease severity in patients with cancer in Wuhan, China: A multicentre, retrospective, cohort study. Lancet Oncol..

[3] Yang F., Shi S., Zhu J., Shi J., Dai K., Chen X. (2020). Clinical characteristics and outcomes of cancer patients with COVID-19. J. Med. Virol..

[4] Calabrò L., Peters S., Soria J.-C., Di Giacomo A.M., Barlesi F., Covre A., Altomonte M., Vegni V., Gridelli C., Reck M. (2020). Challenges in lung cancer therapy during the COVID-19 pandemic. Lancet Respir. Med..

[5] Yu J., Ouyang W., Chua M.L.K., Xie C. (2020). SARS-CoV-2 Transmission in Patients with Cancer at a Tertiary Care Hospital in Wuhan, China. JAMA Oncol..

[6] Lamarre J., Banerjee S., Cervantes A., Garassino M., Garrido P., Girard N., Haanen J., Jordan K., Lordick F., Machiels J. (2020). Managing cancer patients during the COVID-19 pandemic: An ESMO multidisciplinary expert consensus. Ann. Oncol..

[7] Perera R.A.P.M., Tso E., Tsang O.T.Y., Tsang D.N.C., Fung K., Leung Y.W.Y., Chin A.W.H., Chu D.K.W., Cheng S.M.S., Poon L.M.L. (2020). SARS-CoV-2 Virus Culture and Subgenomic RNA for Respiratory Specimens from Patients with Mild Coronavirus Disease. Emerg. Infect. Dis..

[8] World Health Association (2020). Antigen-Detection in the Diagnosis of SARS-CoV-2 Infection Using Rapid Immunoassays. Interim Guidance. https://www.who.int/publications/i/item/antigen-detection-in-the-diagnosis-of-sars-cov-2infection-using-rapid-immunoassays.

[9] Bullard J., Dust K., Funk D., Strong J.E., Alexander D., Garnett L., Boodman C., Bello A., Hedley A., Schiffman Z. (2020). Predicting Infectious Severe Acute Respiratory Syndrome Coronavirus 2 from Diagnostic Samples. Clin. Infect. Dis..

[10] Avanzato V.A., Matson M.J., Seifert S.N., Pryce R., Williamson B.N., Anzick S.L., Barbian K., Judson S.D., Fischer E.R., Martens C. (2020). Case Study: Prolonged Infectious SARS-CoV-2 Shedding from an Asymptomatic Immunocompromised Individual with Cancer. Cell.

[11] Corman V.M., Landt O., Kaiser M., Molenkamp R., Meijer A., Chu D.K., Bleicker T., Brünink S., Schneider J., Schmidt M.L. (2020). Detection of 2019 novel coronavirus (2019-nCoV) by real-time RT-PCR. Eurosurveillance.

[12] Gautret P., Lagier J.C., Parola P., Hoang V.T., Meddeb L., Mailhe M., Doudier B., Courjon J., Giordanengo V., Vieira V.E. (2020). Hydroxychloroquine and azithromycin as a treatment of COVID-19: Results of an open-label non-randomized clinical trial. Int. J. Antimicrob. Agents.

[13] Ramakrishnan M.A. (2016). Determination of 50% endpoint titer using a simple formula. World J. Virol..

[14] Moujaess E., Kourie H.R., Ghosn M. (2020). Cancer patients and research during COVID-19 pandemic: A systematic review of current evidence. Crit. Rev. Oncol..

[15] Addeo A., Friedlaender A. (2020). Cancer and COVID-19: Unmasking their ties. Cancer Treat. Rev..

[16] Weinkove R., McQuilten Z.K., Adler J., Agar M.R., Blyth E., Cheng A.C., Conyers R., Haeusler G.M., Hardie C., Jackson C. (2020). Managing haematology and oncology patients during the COVID -19 pandemic: Interim consensus guidance. Med. J. Aust..

[17] Sun J., Xiao J., Sun R., Tiang X., Liang C., Lin H., Zeng L., Hu J., Yuan R., Zhou P. (2020). Prolonged Persistence of SARS-CoV-2 RNA in Body Fluids. Emerg. Infect. Dis..

[18] Xiao A.T., Tong Y.X., Zhang S. (2020). False negative of RT-PCR and prolonged nucleic acid conversion in COVID-19: Rather than recurrence. J. Med. Virol..

[19] Walsh K.A., Spillane S., Comber L., Cardwell K., Harrington P., Connell J., Teljeur C., Broderick N., de Gascun C.F., Sith S.M. (2020). The duration of infectiousness of individuals infected with SARS-CoV-2. J. Infect..

[20] Strong J.E., Feldmann H. (2017). The Crux of Ebola Diagnostics. J. Infect. Dis..

[21] Gosain R., Abdou Y., Singh A., Rana N., Puzanov I., Ernstoff M.S. (2020). COVID-19 and Cancer: A Comprehensive Review. Curr. Oncol. Rep..

[22] La Scola B., Le Bideau M., Andreani J., Hoang V.T., Grimaldier C., Colson P., Gautret P., Raoult D. (2020). Viral RNA load as determined by cell culture as a management tool for discharge of SARS-CoV-2 patients from infectious disease wards. Eur. J. Clin. Microbiol. Infect. Dis..

[23] European Centre for Disease Prevention and Control (2020). Options for the Use of Rapid Antigen Tests for COVID-19 in the EU/EEA and the UK.

[24] Yongchen Z., Shen H., Wang X., Shi X., Li Y., Yan J., Chen Y., Gu B. (2020). Different longitudinal patterns of nucleic acid and serology testing results based on disease severity of COVID-19 patients. Emerg. Microbes Infect..

[25] World Health Organization (2020). Criteria for releasing COVID-19 patients from isolation. Scientific brief. https://apps.who.int/iris/bitstream/handle/10665/332451/WHO-2019-nCoV-Sci_Brief-Discharge_From_Isolation-2020.1-eng.pdf.

[26] Filippi A.R., Russi E., Magrini S.M., Corvò R. (2020). Letter from Italy: First practical indications for radiation therapy departments during COVID-19 outbreak. Int. J. Radiat. Oncol. Biol. Phys..

